# Assessment of the Knowledge, Attitudes, and Practices of Indian Medical Students Towards Coronavirus Disease 2019 (COVID-19) After Two Doses of Vaccination and Their Approach Towards the Third (Booster) Dose

**DOI:** 10.7759/cureus.55588

**Published:** 2024-03-05

**Authors:** Saptarshi Banerjee, Kumar Sarvottam, Ashish Kumar Gupta

**Affiliations:** 1 Department of Physiology, Institute of Medical Sciences, Banaras Hindu University, Varanasi, IND; 2 Department of Physiology, All India Institute of Medical Sciences, Gorakhpur, IND

**Keywords:** attitudes, covid-19, covaxin, covishield, booster dose, third dose, vaccine hesitancy, practices, knowledge, medical students

## Abstract

Background and objectives

Medical students not only directly impact coronavirus disease 2019 (COVID-19) transmission due to their behavior and perceptions but also play an important role in influencing the behavior and vaccine intentions of their families and the community at large. The study's objective was to assess the knowledge, attitudes, and practices of medical students who have completed two doses of the COVID-19 vaccine towards the disease and their approach towards the third (booster) dose.

Methods

A total of 705 individual responses were obtained from a cross-sectional web-based study deployed using Google Forms. After getting consent and basic information, data was obtained regarding knowledge of the disease, attitudes towards the disease, and practices regarding the same. The mean score was calculated for the above different categories and compared with their respective cut-offs using a one-sample t-test. Data was also collected regarding their approach towards the booster dose of the COVID-19 vaccine, and the proportion of each response for different categories of questions was calculated.

Results

Participants were found to have mean scores in the range of moderate knowledge in the first part (47.67±4.49) and the second part (6.96±1.10) of the questionnaire and moderate practices (30.6±4.27) regarding COVID-19 disease. However, they had a mean score in the range of low attitude (39.79±4.07). The majority of participants acknowledge the role of vaccines in preventing the severity and spread of the disease (71.95%) and its effect on workers and medical professionals (60.26%). Mixed opinions were obtained for concerns regarding its pre-market testing and adverse effects and the government's vaccination policy.

Interpretation and conclusion

Responses of the medical students obtained in this study were majorly positive and in accordance with pre-vaccination studies concerning knowledge and practices. However, the low mean score in attitude obtained can be possibly explained due to their lack of direct exposure to patient management during the pandemic. The majority of participants had a positive response towards the use of the vaccine and the effectiveness of the booster dose, but concerns regarding pre-market testing, adverse effects, and the government's vaccination policy suggested the role of awareness campaigns and government endeavors to curb the same.

## Introduction

The coronavirus disease 2019 (COVID-19) has affected many spheres of human life, including health, education, and the economy, since its emergence [1]. On January 30, 2020, the World Health Organization (WHO) declared the outbreak a Public Health Emergency of International Concern, and on March 11, 2020, it was declared a pandemic. The response of any country to a pandemic is a complex process guided by several factors, and India, as a nation, has stood firmly to manage this challenge with both governmental and non-governmental support [2]. As part of control measures against COVID-19, two vaccines were approved and launched in India: Covishield and Covaxin [3]. An observed decline in humoral immunity after six months of vaccination with the second dose, along with a rise of several COVID-19 variants, called for a booster dose to mediate an increase in immunity and enhance the vaccine's effectiveness [4,5].

The transmission of COVID-19 is hugely dependent on human behavior and perception of the disease, so the study of knowledge, attitudes, and practices is essential [6]. Medical students play a critical role in this regard by influencing the vaccination intention and COVID-responsive behavior of their families and communities; hence, knowing their response to this pandemic is important [7]. Though a considerable number of studies were performed in many nations to assess the knowledge, attitudes, and practices of the general population towards this disease, little data is available for the same among medical students before the administration of vaccines.

It has been reported that 56.6% of healthcare workers and medical students had poor knowledge about COVID-19 and only 46% of the total study sample had positive perceptions of COVID-19. Around 81.7% of them prioritized hand hygiene, but only 73.4% wore masks to prevent COVID-19 [8]. However, a cross-sectional study conducted among medical students in Jordan showed good levels of knowledge, positive attitudes, and good precautionary measures in participants [9]. Similar results were observed in neighboring Asian countries like Iran and Pakistan [10,11].

In India, few studies have explored the knowledge, attitudes, and practices of medical students regarding COVID-19 before the vaccination drive. In a study conducted among medical students, participants were seen to have satisfactory knowledge regarding symptoms, mode of spread, manifestation, and precautions, but knowledge regarding WHO recommendations for the type of mask (31%) and breastfeeding by COVID-19-positive mothers (43%) has not been up to the mark. A high percentage of students showed a positive response in terms of attitude as well as practices, whereas another study showed 92.7% of participants had extensive knowledge, but half of the total participants agreed that ordinary residents can wear general medical masks to prevent infection transmission [12]. In addition, 80% of participants had a positive attitude, and a majority had health-seeking behavioral intentions and prevention practices [13]. Similar findings were obtained in another study, but 18% of them had partial knowledge about the symptoms of severe COVID-19 [14].

With the commencement of the vaccination campaign, over the period, it became evident that COVID-19 transmission can be interrupted by achieving herd immunity [15]. A decreasing trend in mortality, morbidity, and severity of the disease may lead to a change in behavior and perception towards the disease. However, there is a paucity of scientific information assessing the parameters of knowledge, attitudes, and practices in medical students after the completion of two doses of vaccination.

Vaccine hesitancy is perceived as an obstacle to curbing the COVID-19 pandemic, and limited data is available from studies from around the world to assess vaccine hesitancy for booster doses in medical students. Around 74.5% of participants were found to favor the COVID-19 booster dose, whereas 17.6% rejected it and 7.9% were uncertain, as observed in a study done in Poland [5]. A similar study in Japan showed the willingness of 84.5% of participants to receive a third dose [16]. There are no studies done in India aimed at assessing vaccine hesitancy in medical students towards the booster dose. So, in this study, we have conducted a questionnaire-based survey to assess knowledge, attitudes, and practices towards COVID-19 post-vaccination (two doses) and vaccine hesitancy towards the booster dose among undergraduate medical students enrolled in Bachelor of Medicine and Bachelor of Surgery (MBBS) courses at various medical colleges in India.

## Materials and methods

A total of 705 individual responses were obtained from all over India for the study conducted over two months, from the end of June 2022 to September 2022. MBBS students (first year to fourth year) of age group 18-30, belonging to either gender, who had completed two doses of vaccination (Covishield or Covaxin) were included. In contrast, any MBBS student who had not completed two doses of vaccine or who did not wish to participate in the study was excluded.

For this cross-sectional web-based study, an anonymous online structured questionnaire was developed using the questionnaire of WHO training material for the detection, prevention, response, and control of COVID-19 and questionnaires based on COVID-19 vaccine hesitancy among medical students [3,17,18]. We have used a pre-validated questionnaire with good internal consistency for determining the knowledge, attitudes, and practices of the students (Cronbach alpha=0.73). To assess vaccine hesitancy, we developed a draft questionnaire which was pretested among 40 medical students (Cronbach alpha=0.71) for clarity and acceptability, and modifications were made accordingly in the final questionnaire. The questionnaire was deployed using Google Forms. Its link was shared within the social media network of medical students both individually and through email and WhatsApp groups, which was further circulated by the students, who could further click on the link to view and answer the questions.

English was chosen as the preferred language as it is the medium of instruction of medical courses throughout India, and changes were made as required to enable a better understanding of the questions. A consent form and information sheet (Appendix A) was included consisting of a short introduction regarding objectives, procedures, the voluntary nature of participation, declarations of confidentiality, and anonymity. It also contained questions regarding age, gender, name, and place of college.

The questionnaire (Appendix B) consisted of four sections: knowledge about COVID-19, attitudes towards COVID-19, practices regarding COVID-19, and approach towards booster dose of COVID-19 vaccine [3,17].

The knowledge section consisted of 26 questions in two parts: The first part of knowledge had 18 questions, of which three sets of six questions each were regarding characteristics, symptoms, and prevention or control of the disease, respectively. The questions were either in the form of multiple-choice single answers or in the form of true, false, or no opinion carrying individual scores (Appendix C). The score obtained in the first part of knowledge denoted as "KF" was ranged and categorized into low, moderate, and high knowledge (Appendix D). The second part of knowledge had eight questions with four each regarding the route of transmission and potentially high-risk groups. The questions were in the form of true or false with true response carrying 1 point and false carrying 0 point. The score obtained in the second part of knowledge denoted as "KS" was ranged and categorized as shown in Appendix E.

There were 15 questions to evaluate the attitudes of the medical students after taking two doses of vaccination. The questions were in the form of true, false, and no opinion with the scoring system the same as above. The score obtained in attitudes denoted as "A" was ranged and categorized as shown in Appendix F.

Regarding the practice of medical students after two doses of vaccination, 12 questions were asked (the scoring system the same as above) with the score obtained in practice denoted as "P" ranged and categorized as shown in Appendix G.

Regarding the approach of medical students towards the third (booster) dose of vaccine, nine questions were there, of which two questions were regarding the role of the vaccine in preventing severity and spread (H1-H2), four questions evaluated the concerns regarding the vaccine (H3-H6), and three questions were related to its effect on health worker/medical professional (H7-H9). The questions were in the form of multiple-choice single answers with options based on a 5-point Likert scale (strongly disagree, disagree, neither disagree nor agree, agree, strongly agree) (Appendix H and Appendix I).

The scores for each of the categories, namely, the first part of knowledge (KF), the second part of knowledge (KS), attitudes (A), and practice (P), were assigned using the abovementioned scoring system, and the percentage of responses corresponding to each category was calculated. The data obtained was compiled in a spreadsheet, and a one-sample t-test was performed to compare the mean score in "KF," "KS," "A," and "P" against their respective cut-offs. The percentage of responses for each option of the Likert scale in "Approach towards booster dose of COVID-19 vaccine" was tabulated and depicted in pie charts, to know the proportion of each response for each of the three categories of questions. All the data was analyzed by IBM SPSS Statistics for Windows, Version 23.0 (Released 2015; IBM Corp., Armonk, New York, United States).

The study was approved by the Ethical Committee of the Institute of Medical Sciences, Banaras Hindu University (approval number: Dean/2022/EC/3334), and recommendations of the Helsinki Declaration were followed while recording the data. No financial or in-kind reward was given to students who completed the survey.

## Results

The percentage of responses corresponding to each category for different parameters of the knowledge, attitude, and practice study is compiled below (Table 1).

**Table 1:** Percentage of responses corresponding to each category for different parameters of the knowledge, attitude, and practice study KF: score obtained in the section of the first part of knowledge; KS: score obtained in the section of the second part of knowledge; A: score obtained in the section of attitude; P: score obtained in the section of practice

Parameter	Category	Number of responses (out of 705)	Percentage of responses
KF	Low knowledge	212	30.1%
	Moderate knowledge	341	48.3%
	High knowledge	152	21.5%
KS	Low knowledge	24	3.4%
	Moderate knowledge	477	67.6%
	High knowledge	204	28.9%
A	Low attitude	235	33.3%
	Moderate attitude	415	58.8%
	High attitude	55	7.8%
P	Weak practice	181	25.7%
	Moderate practice	484	68.6%
	High practice	40	5.6%

Participants had a mean score in the range of moderate knowledge in the section of the first part of knowledge with 493 of them (69.8%) having high to moderate knowledge (Table 2).

**Table 2:** Comparison between the mean score of the first part of knowledge and cut-off values KF: score obtained in the section of the first part of knowledge

KF (mean±standard deviation)	Cut-off values	
	46	50
47.67±4.49	t(704)=9.86, p=0.00	t(704)=-13.74, p=0.00

Six hundred and eighty-one of them (96.5%) had high to moderate knowledge in the section of the second part of knowledge with a mean score in the range of moderate knowledge (Table 3).

**Table 3:** Comparison between the mean score of the second part of knowledge and cut-off values KS: score obtained in the section of the second part of knowledge

KS (mean±standard deviation)	Cut-off values	
	5	8
6.96±1.10	t(704)=47.24, p=0.00	t(704)=-24.95, p=0.00

Around 92.1% of participants (650 responses) showed a low to moderate attitude towards COVID-19. The mean score of "A" was found in the range of low attitude (Table 4).

**Table 4:** Comparison between the mean score of attitude and cut-off values A: score obtained in the section of attitude

A (mean±standard deviation)	Cut-off values	
	40	43
39.79±4.07	t(704)=-1.78, p=0.00	t(704)=-21.34, p=0.00

Around 74.2% of participants (524 responses) had moderate to high practices regarding COVID-19. The mean score of "P" for all participants was found to be in the category of moderate practice (Table 5).

**Table 5:** Comparison between the mean score of practice and cut-off values P: score obtained in the section of practice

P (mean±standard deviation)	Cut-off values	
	29	34
30.6±4.27	t(704)=10.14, p=0.00	t(704)=-20.90, p=0.00

Responses corresponding to each category of questions assessing the approach of medical students towards the third dose are compiled below.

**Figure 1:**
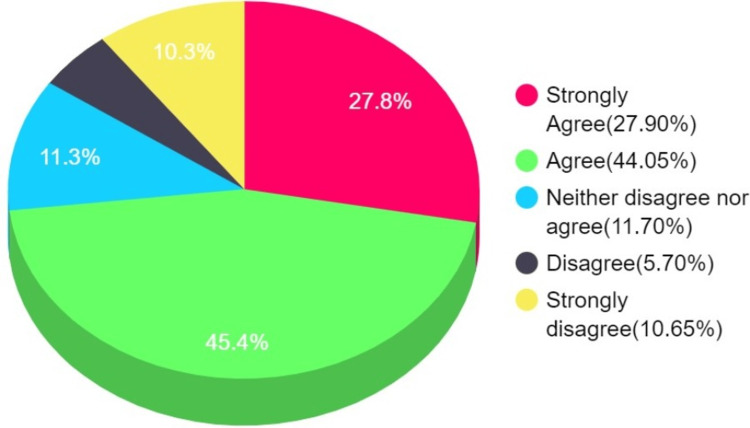
Concerns regarding the role of the vaccine in preventing the severity and spread of the disease (H1-H2)

The majority of participants (strongly agree and agree sections of Figure 1) think the vaccine has a role in preventing the severity and spread of the disease (Figure 1).

**Figure 2:**
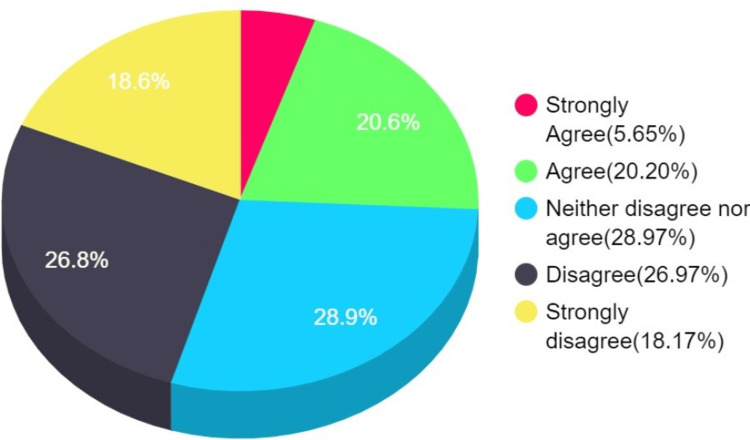
Concerns regarding the vaccine (H3-H6)

Participants have mixed opinions for concerns regarding vaccines about their pre-market testing, their adverse effects, and the government's vaccination policy (Figure 2).

**Figure 3:**
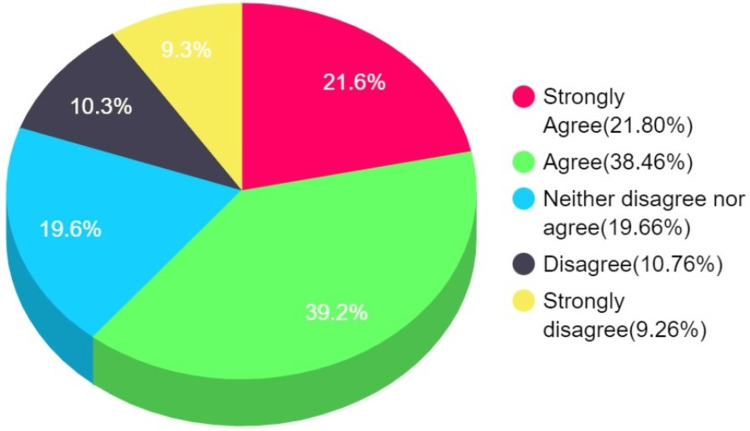
Concerns regarding the vaccine's effect on health workers/medical professionals (H7-H9)

Participants have a majorly affirming response (strongly agree and agree sections of Figure 3) acknowledging the vaccine's effect on health workers and medical professionals (Figure 3).

## Discussion

The knowledge, attitudes, and practices for a particular disease are influenced by various factors, namely, the gravity of the illness, the severity of its spread, and the fatality rate [19]. This study was started with the assumption that the current decreasing trend in mortality, morbidity, and severity of COVID-19 along with the completion of two doses of vaccination might affect the knowledge, attitudes, and practices of medical students towards the disease. The majority of participants were found to have sufficient knowledge regarding the characteristics and symptoms of the disease as well as regarding the prevention and control of the disease. However, about 30% of them were found to have low knowledge about the same. Almost all of them had adequate knowledge regarding the route of transmission and higher-risk groups. More than 90% of them had a moderate to low attitude towards COVID-19. The mean score was in the range of low attitude. It was noteworthy that three-fourths of them were proactive regarding proper practices for the prevention of COVID-19.

The low mean score in attitude obtained can be explained by the fact that medical students up to their final year were not directly exposed to patient management and government awareness programs as they were not first-line healthcare workers. Their clinical postings started only when the situation was slightly under control and a majority of their knowledge was social media and print media driven, thereby misjudging the gravity of the problem [8,14].

In a similar study by Gohel et al., before the vaccination drive, it was shown that about 40% of participants had incorrect or partial knowledge regarding the symptomatology of COVID-19, while half of them couldn't correctly identify the modes of transmission. About 20% of participants did not agree that wearing a surgical mask could prevent COVID-19. The rest of them showed a positive perception towards COVID-19 prevention and control [14]. Another study done in Rajasthan showed students had insufficient knowledge regarding the type of mask advised by WHO (31%) and the indication of breastfeeding in COVID-19-positive mothers (43%) [12]. However, more than 90% of participants had requisite knowledge about the symptoms and mode of transmission, and only 11% opined not to wear a mask in our study. Around 83% of them had ample knowledge about the effect of COVID-19 on pregnant women.

A study done on students of the Government Doon Medical College showed adequate knowledge of the participants about the disease and similar attitudes and practices [13]. Similar results were also obtained in studies conducted in Jordan, Iran, and Pakistan [9,12,13]. No significant data was available to the best of our knowledge regarding the same parameters post-vaccination for us to compare.

According to the WHO Strategic Advisory Group of Experts (SAGE) group, "vaccine hesitancy refers to a delay in acceptance or refusal of vaccination despite the availability of vaccination services" [20]. In our study, the role of the vaccine in preventing the severity and spread of the disease was acknowledged by the majority (about 70%) of the participants, whereas about 12% were indecisive, and 16% disagreed with the fact. Regarding pre-market testing, adverse effects, and the government's vaccination policy, about 26% of them expressed negative concern, and about 45% of them disagreed with having any sort of concern, while 29% of them preferred to remain neutral. About 60% of them agreed with the fact that vaccination is essential for healthcare workers. Studies done in Poland and Japan revealed that 74.5% and 84.5% of medical students were willing to receive the third dose, respectively [5,16]. Studies in the general population to assess booster dose hesitancy revealed that only 50% of respondents were willing to take the booster dose [21].

The willingness of students to receive the booster dose is found to be greater than that of the normal population, suggesting their greater awareness regarding the disease. However, medical students in nations like Japan and Poland were more willing to receive the vaccine, thereby suggesting the role of government in initiating programs directed towards encouraging the general population, and particularly medical students, to receive the vaccine.

This study, however, did not explore the causes that could lead to a low attitude among medical students. The association among different knowledge, attitude, and practice parameters and between different knowledge, attitude, and practice parameters and socio-demographic characteristics are also not evaluated. In addition, online surveys could lead to bias in measurement as students had access to the internet all the time while responding to the questionnaire.

Thus, this study opens up a vast avenue for future research in terms of identifying the causes of low attitude and finding the association among different knowledge, attitude, and practice parameters and between different knowledge, attitude, and practice parameters and socio-demographic parameters. Causes leading to concerns about the vaccine among medical students can also be explored further.

## Conclusions

The majority of the responses of medical students corresponding to knowledge and practices were positive and in line with similar studies done pre-vaccination, with noteworthy improvement in knowledge regarding symptoms, routes of transmission, high-risk groups, and mask-wearing practices. However, a low mean score in attitude asserts enforcement of the awareness program among medical students.

The participants were found to be majorly affirmative in terms of the use of the vaccine and the effectiveness of the booster dose, but concerns were noted regarding pre-market testing, adverse effects, and the government's vaccination policy. The willingness of the students to receive the booster dose was found to be greater than that of the general population. However, the willingness of medical students to receive the third dose in India was found to be lower than that of nations like Poland and Japan, and their concerns regarding the vaccine suggest the requirement of the government's positive endeavor to spread awareness and increase the willingness of people, especially medical students, regarding booster dose.

## Appendices

Appendix A

**Table 6:** Consent form and information sheet

Consent form and information sheet
This is an online questionnaire developed to assess the knowledge, attitudes, and practices towards COVID-19 disease and the approach towards third (booster) dose in medical students who have completed two doses of COVID-19 vaccine. MBBS students are requested to participate in this study in which they have to answer the following questions. Participation of the student is voluntary, and he/she may refuse to participate or withdraw from the study at any time. The information concerning the participation of patients in this study will be kept confidential to the full extent permitted by law and used only for scientific purposes. The patient's name will not be used in any reports released anyway. Proceed ahead if you wish to participate.
Name:
Email ID:
Age:
Gender:
Full name of the college:
State in which the college is situated:

Appendix B

**Table 7:** Questionnaire *: indicator of the correct answer

Questionnaire		
Knowledge about COVID-19		
First part of knowledge		
K1	I have heard about COVID-19	True*
		False
		No opinion
K2	COVID-19 is a contagious disease	True*
		False
		No opinion
K3	Which of the following is the cause of COVID-19?	Bacteria
		Virus*
		Fungi
		Parasite
		Immunodeficiency
		No opinion
K4	How long is the incubation period of the disease?	Less than 2 days
		2-5 days
		3-14 days*
		No opinion
K5	Which of the following is the treatment for COVID-19?	Symptomatic therapy*
		Antibiotics
		No treatment
		No opinion
K6	In which age group is the disease more dangerous?	Under 15 years
		15-30 years
		30-50 years
		Above 50 years*
		No opinion
K7	Fever is a symptom of COVID-19	True*
		False
		No opinion
K8	Cough is a symptom of COVID-19	True*
		False
		No opinion
K9	Sore throat is a symptom of COVID-19	True*
		False
		No opinion
K10	Body pain is a symptom of COVID-19	True*
		False
		No opinion
K11	Diarrhea or constipation is a symptom of COVID-19	True*
		False
		No opinion
K12	Headache is a symptom of COVID-19	True*
		False
		No opinion
K13	In suspecting infection with COVID-19, primarily I will measure fever	True*
		False
		No opinion
K14	In suspecting infection with COVID-19, primarily I will visit a physician	True
		False*
		No opinion
K15	In suspecting infection with COVID-19, I will avoid unnecessary daily activities	True*
		False
		No opinion
K16	To avoid contracting COVID-19, I will avoid contact with individuals suspected to be infected with COVID-19	True*
		False
		No opinion
K17	The prevalence of COVID-19 disease is increasing in India	True*
		False
		No opinion
K18	Washing hands with water and soap can eliminate the causes of the disease	True*
		False
		No opinion
Second part of knowledge		
K19	The disease can be transmitted directly through cough	True*
		False
K20	The disease can be transmitted directly through contact with infected surfaces	True*
		False
K21	The disease can be transmitted directly through the consumption of contaminated dairy and meat	True
		False*
K22	The disease can be transmitted directly through contact with infected individuals (handshaking, hugging, kissing)	True*
		False
K23	The disease is more dangerous in pregnant women	True*
		False
K24	The disease is more dangerous in old individuals	True*
		False
K25	The disease is more dangerous in people with weakened immune systems	True*
		False
K26	The disease is more dangerous in people with cancer, diabetes, and chronic respiratory diseases	True*
		False
Attitude towards COVID-19		
A1	It is my opinion that early detection of COVID-19 can improve treatment and outcome	True*
		False
		No opinion
A2	It is my opinion that COVID-19 can be treated at home	True*
		False
		No opinion
A3	It is my opinion that health education can help prevent COVID-19	True*
		False
		No opinion
A4	It is my opinion that COVID-19 is a serious disease	True*
		False
		No opinion
A5	It is my opinion that COVID-19 can be avoided by proper precautions	True*
		False
		No opinion
A6	It is my opinion that if there is an available vaccine for the disease, it should be used	True*
		False
		No opinion
A7	It is my opinion that COVID-19 is a curable disease	True*
		False
		No opinion
A8	It is my opinion that the awareness of COVID-19 disease in society is sufficient	True*
		False
		No opinion
A9	It is my opinion that COVID-19 disease results in death in all cases	True
		False*
		No opinion
A10	It is my opinion that COVID-19 disease can be transmitted through household pets to humans	True
		False*
		No opinion
A11	It is my opinion that authorities should restrict travel to and from COVID-19 disease areas to prevent contamination	True*
		False
		No opinion
A12	It is my opinion that authorities should quarantine COVID-19 patients in special hospitals	True*
		False
		No opinion
A13	It is my opinion that in the event of an increase in the number of cases of COVID-19, authorities should be ready to close educational centers (kindergartens, schools, and universities)	True*
		False
		No opinion
A14	It is my opinion that authorities should be prepared to restrict access to religious sites, shrines, and mosques if the number of COVID-19 cases increases	True*
		False
		No opinion
A15	It is my opinion that if the number of COVID-19 cases increases, authorities should be ready to lock down and quarantine the city	True*
		False
		No opinion
Practices regarding COVID-19		
P1	In order to prevent contracting and spreading COVID-19, I avoid going out of my home	True*
		False
		No opinion
P2	In order to prevent contracting and spreading COVID-19, I avoid unnecessary vacations	True*
		False
		No opinion
P3	In order to prevent contracting and spreading COVID-19, I avoid consuming outdoor food	True*
		False
		No opinion
P4	In order to prevent contracting and spreading COVID-19, I avoid handshaking, hugging, and kissing	True*
		,False
		No opinion
P5	In order to prevent contracting and spreading COVID-19, I avoid public transportation (taxi, bus, subway, plane, train)	True*
		False
		No opinion
P6	In order to prevent contracting and spreading COVID-19, I avoid going to work	True*
		False
		No opinion
P7	In order to prevent contracting and spreading COVID-19, I frequently wash my hands	True*
		False
		No opinion
P8	In order to prevent contracting and spreading COVID-19, I pay more attention to my personal hygiene than usual	True*
		False
		No opinion
P9	In order to prevent contracting and spreading COVID-19, I use disinfectant and solutions	True*
		False
		No opinion
P10	In order to prevent contracting COVID-19, I use herbal products and traditional medicine	True*
		False
		No opinion
P11	In order to prevent contracting COVID-19, I take vitamin supplements	True*
		False
		No opinion
P12	In order to prevent contracting and spreading COVID-19, when do you use facial masks?	Never
		Only in public and crowded places*
		Most of the time
		Always
		No opinion
Approach towards booster dose of COVID-19 vaccine		
H1	"COVID-19 vaccine can reduce the spread of the disease in the community"	Strongly disagree
		Disagree
		Neither disagree nor agree
		Agree
		Strongly agree*
H2	"COVID-19 vaccine can help reduce severe COVID-19 disease"	Strongly disagree
		Disagree
		Neither disagree nor agree
		Agree
		Strongly agree*
H3	"I disagree with the choice of vaccine or vaccination recommendation provided by my government"	Strongly disagree*
		Disagree
		Neither disagree nor agree
		Agree
		Strongly agree
H4	"I am concerned that the present COVID-19 vaccine booster doses are not effective enough"	Strongly disagree*
		Disagree
		Neither disagree nor agree
		Agree
		Strongly agree
H5	"I am concerned about the serious adverse events from the currently available COVID-19 vaccine booster dose"	Strongly disagree*
		Disagree
		Neither disagree nor agree
		Agree
		Strongly agree
H6	"I am concerned that the present COVID-19 vaccine booster dose might not have been tested rigorously prior to launch"	Strongly disagree*
		Disagree
		Neither disagree nor agree
		Agree
		Strongly agree
H7	"I trust the information I am receiving about the COVID-19 vaccine from the government or public health experts"	Strongly disagree
		Disagree
		Neither disagree nor agree
		Agree
		Strongly agree*
H8	"COVID-19 booster dose should be made mandatory for healthcare worker"	Strongly disagree
		Disagree
		Neither disagree nor agree
		Agree
		Strongly agree*
H9	"Apart from the COVID-19 vaccine, I have also received other vaccine(s) after joining as a medical student"	Strongly disagree
		Disagree
		Neither disagree nor agree
		Agree
		Strongly agree*

Appendix C

**Table 8:** Scoring system of the first part of knowledge

Scoring system of the first part of knowledge	
Correct answer	3 points
Incorrect answer	1 point
"No opinion" answer	2 points

Appendix D

**Table 9:** Categories corresponding to the range of scores for KF KF: first part of knowledge

Score	Category
46 and under	Low knowledge
47-50	Moderate knowledge
Above 50	High knowledge

Appendix E

**Table 10:** Categories corresponding to the range of scores for KS KS: second part of knowledge

Score	Category
Less than 5	Low knowledge
5-7	Moderate knowledge
8	High knowledge

Appendix F

**Table 11:** Categories corresponding to the range of scores for A A: attitude

Score	Category
Under 40	Low attitude
40-43	Moderate attitude
44-45	High attitude

Appendix G

**Table 12:** Categories corresponding to the range of scores for P P: practice

Score	Category
Under 29	Weak practice
29-34	Moderate practice
Greater than 34	High practice

Appendix H

**Table 13:** Scoring criteria for vaccine hesitancy for questions H3-H6

Scoring criteria for vaccine hesitancy for questions H3-H6	
Strongly disagree	5 points
Disagree	4 points
Neither disagree nor agree	3 points
Agree	2 points
Strongly agree	1 point

Appendix I

**Table 14:** Scoring criteria for vaccine hesitancy for the rest of the questions (except H3-H6)

Scoring criteria for vaccine hesitancy for the rest of the questions (except H3-H6)	
Strongly disagree	1 point
Disagree	2 points
Neither disagree nor agree	3 points
Agree	4 points
Strongly agree	5 points

## References

[1] Crayne MP (2020). The traumatic impact of job loss and job search in the aftermath of COVID-19. Psychol Trauma.

[2] Radhakrishnan N, Gupta DK (2021). India's COVID-19 response: science first. Lancet.

[3] Jain J, Saurabh S, Kumar P (2021). COVID-19 vaccine hesitancy among medical students in India. Epidemiol Infect.

[4] Levin EG, Lustig Y, Cohen C (2021). Waning immune humoral response to BNT162b2 Covid-19 vaccine over 6 months. N Engl J Med.

[5] Dziedzic A, Issa J, Hussain S (2022). COVID-19 vaccine booster hesitancy (VBH) of healthcare professionals and students in Poland: cross-sectional survey-based study. Front Public Health.

[6] Morens DM, Fauci AS (2020). Emerging pandemic diseases: how we got to COVID-19. Cell.

[7] Jain L, Vij J, Satapathy P (2021). Factors influencing COVID-19 vaccination intentions among college students: a cross-sectional study in India. Front Public Health.

[8] Bhagavathula AS, Aldhaleei WA, Rahmani J, Khubchandani J (2020). Knowledge, attitude, perceptions and practice towards COVID-19: a systematic review and meta-analysis [PREPRINT]. medRxiv.

[9] Khasawneh AI, Humeidan AA, Alsulaiman JW (2020). Medical students and COVID-19: knowledge, attitudes, and precautionary measures. A descriptive study from Jordan. Front Public Health.

[10] Taghrir MH, Borazjani R, Shiraly R (2020). COVID-19 and Iranian medical students; a survey on their related-knowledge, preventive behaviors and risk perception. Arch Iran Med.

[11] Noreen K, Rubab ZE, Umar M, Rehman R, Baig M, Baig F (2020). Knowledge, attitudes, and practices against the growing threat of COVID-19 among medical students of Pakistan. PLoS One.

[12] Joshi R, Takhar R, Jain S (2021). Knowledge, attitude and practices associated with COVID-19 among undergraduate medical students of Rajasthan. Int J Community Med Public Health.

[13] Maheshwari S, Gupta PK, Sinha R, Rawat P (2020). Knowledge, attitude, and practice towards coronavirus disease 2019 (COVID-19) among medical students: a cross-sectional study. J Acute Dis.

[14] Gohel KH, Patel PB, Shah PM, Patel JR, Pandit N, Raut A (2021). Knowledge and perceptions about COVID-19 among the medical and allied health science students in India: an online cross-sectional survey. Clin Epidemiol Glob Health.

[15] Kadkhoda K (2021). Herd immunity to COVID-19. Am J Clin Pathol.

[16] Sugawara N, Yasui-Furukori N, Fukushima A, Shimoda K (2021). Attitudes of medical students toward COVID-19 vaccination: who is willing to receive a third dose of the vaccine?. Vaccines (Basel).

[17] Erfani A, Shahriarirad R, Ranjbar K, Mirahmadizadeh A, Moghadami M (2023). Knowledge, attitude, and practice toward the novel coronavirus (COVID-19) outbreak: a population-based survey in Iran. J Health Sci Surveill Syst.

[18] Chandani S, Jani D, Sahu PK (2021). COVID-19 vaccination hesitancy in India: state of the nation and priorities for research. Brain Behav Immun Health.

[19] Alzoubi H, Alnawaiseh N, Al-Mnayyis A, Abu-Lubad M, Aqel A, Al-Shagahin H (2020). COVID-19 - knowledge, attitude and practice among medical and non-medical university students in Jordan. J Pure Appl Microbiol.

[20] Mangla S, Zohra Makkia FT, Pathak AK (2021). COVID-19 vaccine hesitancy and emerging variants: evidence from six countries. Behav Sci (Basel).

[21] Achrekar GC, Batra K, Urankar Y (2022). Assessing COVID-19 booster hesitancy and its correlates: an early evidence from India. Vaccines (Basel).

